# Connexin43 Hemichannels in Satellite Glial Cells, Can They Influence Sensory Neuron Activity?

**DOI:** 10.3389/fnmol.2017.00374

**Published:** 2017-11-16

**Authors:** Mauricio A. Retamal, Manuel A. Riquelme, Jimmy Stehberg, Julio Alcayaga

**Affiliations:** ^1^Centro de Fisiología Celular e Integrativa, Facultad de Medicina, Clinica Alemana Universidad del Desarrollo, Santiago, Chile; ^2^Department of Cell Physiology and Molecular Biophysics, School of Medicine, Texas Tech University Health Sciences Center, Lubbock, TX, United States; ^3^Department of Biochemistry and Structural Biology, University of Texas Health Science Center, San Antonio, TX, United States; ^4^Laboratorio de Neurobiología, Centro de Investigaciones Biomedicas, Universidad Andres Bello, Santiago, Chile; ^5^Department of Biology, Cell Physiology Center, University of Chile, Santiago, Chile

**Keywords:** gap junction channel, hemichannel, sensory neurons, gliotransmitters, satellite glial cells

## Abstract

In this review article, we summarize the current insight on the role of Connexin- and Pannexin-based channels as modulators of sensory neurons. The somas of sensory neurons are located in sensory ganglia (i.e., trigeminal and nodose ganglia). It is well known that within sensory ganglia, sensory neurons do not form neither electrical nor chemical synapses. One of the reasons for this is that each soma is surrounded by glial cells, known as satellite glial cells (SGCs). Recent evidence shows that connexin43 (Cx43) hemichannels and probably pannexons located at SGCs have an important role in paracrine communication between glial cells and sensory neurons. This communication may be exerted via the release of bioactive molecules from SGCs and their subsequent action on receptors located at the soma of sensory neurons. The glio-neuronal communication seems to be relevant for the establishment of chronic pain, hyperalgesia and pathologies associated with tissue inflammation. Based on the current literature, it is possible to propose that Cx43 hemichannels expressed in SGCs could be a novel pharmacological target for treating chronic pain, which need to be directly evaluated in future studies.

## Hemichannels and Pannexons

It is well accepted that paracrine and autocrine cellular communication are critical for cellular and tissular function. Among the main proteins that play a role in these processes are connexins and pannexins. Connexins are unique transmembrane proteins, because they form two different types of channels; hemichannels and gap junction channels (GJCs). While hemichannels are formed by the oligomerization of six connexins, GJCs are formed by the serial docking of two hemichannels, each one provided by one of the contacting cells. On the other hand, a pannexon is formed by 6 subunits of a transmembrane protein called pannexin. Despite connexins and pannexins being very different in terms of sequence, they share a common plasma membrane topology, which comprises four transmembrane domains, two extracellular loops, one intracellular loop and both the N- and C-terminus facing the cytoplasm (Panchin, 2005). Another difference between connexins and pannexins is that unlike connexins, pannexins are believed to form pannexons only, and not gap junction-like structures (Sosinsky et al., 2011). This may be explained because pannexons (at least those formed by pannexin1 (Panx1) and pannexin3 (Panx3)) are glycosylated (Sosinsky et al., 2011; Penuela et al., 2014), which is believed to block the interaction between pannexons, preventing the docking and formation of gap junction like-structures (Locovei et al., 2006; Huang et al., 2007; Penuela et al., 2007, 2009). However, some studies suggest that the formation of gap junction like-structures composed of pannexins could depend of the cell type. For example, in HeLa cells Panx1 and Panx3 may form GJCs, with different properties compared to those formed by connexins (Bruzzone et al., 2003; Sahu et al., 2014).

At the plasma membrane both hemichannels and pannexons are mostly kept in a closed state to prevent cell lysis (Retamal et al., 2015; García et al., 2016). Because these two type of channels are permeable to ions and large molecules, such as ATP and glutamate (Montero and Orellana, 2015). Moreover, the persistent opening could cause passive loss of ion gradients and metabolites (Retamal et al., 2015). However, the relationship between pannexons and cell death is more complex, because it is well known that P2X7-ATP receptor has a close interaction with Panx1, and hence, pannexon opening can trigger the activation of P2X7 receptors channels (Pelegrin and Surprenant, 2006; Iglesias et al., 2008). Moreover, when Panx1 channels open, the released ATP is able to activate P2X7 receptors, which have been associated with the induction of cell death in several cell types, like T-cells (Shoji et al., 2014), Schwann cells (Luo et al., 2013), tumoral cells (Bian et al., 2013) and astrocytes (Wang et al., 2012) among others.

As mentioned above, under physiological conditions hemichannels and pannexons are mainly closed. However, their low open probability is enough to allow these channels to participate in several cellular functions. Accordingly, open hemichannels allow the flow of molecules such as ATP (Stout et al., 2002), glutamate (Ye et al., 2003), NAD+ (Bruzzone et al., 2001), lactate (Karagiannis et al., 2016), glucose (Retamal et al., 2007) and glutathione (Stridh et al., 2008) through the plasma membrane. Moreover, there are several post-translational modifications that increase hemichannel activity, such as phosphorylation, S-nitrosylation, intracellular Ca^2+^ increments and intracellular reducing redox potential among several others (Retamal et al., 2006, 2007; De Vuyst et al., 2009; Batra et al., 2014), which can increase their opening probability in several processes at physiological conditions. Interestingly, connexins are not only found at the cellular plasma membrane. In fact, connexin43 (Cx43) hemichannels have been detected in the mitochondria and may be important for mitochondrial Ca^2+^ and K^+^ uptake (Boengler et al., 2013; Gadicherla et al., 2017), and in mitochondrial hypoxia/reoxygenation preconditioning (Schulz and Heusch, 2006). Moreover, Cx43 hemichannels have been involved in myocardial cell death (Gadicherla et al., 2017). On the other hand, connexin26 (Cx26) hemichannels participate in light processing in the retina, via extracellular potentials in cones modulated by current flowing through connexin hemichannels at the tips of horizontal cell dendrites (Kamermans and Fahrenfort, 2004). Most importantly for this review, in the central nervous system, hemichannels participate in astrocyte to neuron communication in both physiological and pathological conditions, as has been revised extensively by Cheung et al. (2014) and Orellana (2016).

We would like to point it out that, despite all the advances in the field, knowledge on the pharmacology of hemichannels and pannexons is still limited as it has been recently discussed (Nielsen et al., 2017). Among all hemichannel inhibitors mimetic peptides are probably the most specific and have been used in studies both *in vitro* and *in vivo* (O’Carroll et al., 2013; Abudara et al., 2014) representing nowadays a powerful tool for hemichannel research.

## What Is Gliotransmission?

For several years the dogma in synaptic transmission stated that in chemical synapses “information” was transmitted from the presynaptic to the postsynaptic neuron. However, in the late nineties Araque et al. (1999) introduced the term “tripartite synapse”, which referred to the fact that in addition to the pre and postsynaptic neurons, astrocytes that surround chemical synapses are able to modify the synaptic microenvironment and modulate synaptic transmission. To this end, astrocytes express multiple neurotransmitter receptors which allow them to sense synaptic activity (Orellana and Stehberg, 2014) and release molecules that induce responses in neurons. These transmitters are currently known as gliotransmitters (Montero and Orellana, 2015; Harada et al., 2016).

Among glial cells, astrocytes are possibly the most studied in terms of gliotransmitter release, and among all the molecules that are released from astrocytes (for more details see Moraga-Amaro et al., 2014; Montero and Orellana, 2015), the three gliotransmitters that have the greatest evidence for modulating synapses are glutamate, ATP and D-serine (Giaume et al., 2013; Harada et al., 2016). Astrocytes have several gliotransmitter release mechanisms, such as vesicle-based exocytosis (Jorgacevski et al., 2017), P2X7 receptor (Suadicani et al., 2006), maxi-anion channel (Liu et al., 2008), pannexons (Prochnow et al., 2012) and hemichannels (revised by Orellana et al., 2016).

Astrocytes express mainly Cx43 and Panx1 which form functional hemichannels under both physiological and pathological conditions (Huang et al., 2007; Montero and Orellana, 2015). Recent evidence supports the role of hemichannels and pannexons in the interaction between glial cells and neurons at the central nervous system. For example, antidepressants such as fluoxetine, duloxetine, paroxetine, reboxetine, amitriptyline, imipramine and venlafaxine have been reported to inhibit LPS-induced opening of astrocytic Cx43 hemichannels (Jeanson et al., 2016). This result opens the possibility that Cx43 hemichannels may be involved in depression—at least to some extent. This idea is further supported by evidence showing that chronic stress—a model used to induce depressive-like behaviors in rodents—induces increased Cx43 hemichannel and Panx1 pannexon opening in hippocampal astrocytes, with a concomitant Cx43 dependent increase in extracellular glutamate and ATP (Orellana et al., 2015) Additionally, Cx43 hemichannels have been shown to be required for fear memory consolidation in the basolateral amygdala (Stehberg et al., 2012). In this work, it was suggested that one or more gliotransmitters released through Cx43 hemichannels are critical for memory consolidation (Stehberg et al., 2012). Similarly, pannexons formed by Panx1 also participate in the maintenance of the synaptic strength and plasticity of hippocampal neurons (Prochnow et al., 2012; Ardiles et al., 2014). In conclusion, Cx43 hemichannels and Panx1 pannexons are very important for the fine tuning of the synaptic activity in the central nervous system, and is very likely that this role is accomplished through the release of gliotransmitters such as glutamate, ATP and D-serine.

## Connexins and Pannexins in Sensory Ganglion Cells

As presented above, within the central nervous system, evidence for the participation of connexin and pannexin channels is accumulating (Thompson et al., 2008; Orellana and Stehberg, 2014; Orellana et al., 2016). However, in the peripheral nervous system, information on the role of these proteins in glio-neuronal communication is only beginning to emerge. Probably the first study that suggested that connexins have a role in sensory neuron activity was performed in primary co-cultures of sensory neurons from rat dorsal root ganglia (DRG) and smooth muscle cells. In this study, it was found that sensory neurons expressed mRNAs for Cx40 and Cx43, possibly mediating IP_3_-mediated calcium coupling between those two cell types (Ennes et al., 1999). Those results suggest that at least in tissue culture, connexin-based channels allow sensory neurons to couple metabolically with smooth muscle cells. Then Chen et al. (2002), demonstrated that rat petrosal neurons do not express Cx43 under normal conditions but satellite glial cells (SGCs) do. Interestingly, Cx43 immunoreactivity was detected in neurons after 2 weeks of hypoxia. Similarly, Cx43 has been reported in SGCs of spinal (Procacci et al., 2008) and trigeminal (Ohara et al., 2008) ganglia. Additionally, in another study, neurons and SGCs from the trigeminal ganglion were shown the expression of Cx26, Cx36 and Cx40 (Garrett and Durham, 2008). More recently, the presence of the mRNA of Cx26, Cx37, Cx43, Cx45, Panx 1 and Panx2 in the nodose ganglion was observed (Retamal et al., 2014a). Thus, it is clear that sensory neurons and SGCs have the building blocks for establishing paracrine communication through the release of both neuro- and gliotransmitters. Regardless of how suggestive their presence may be, studies characterizing the structure, composition and functionality of connexin and pannexin channels in these cell types are needed, to understand their function under physiological conditions.

## Connexin and Pannexin -Mediated Glia-To-Neuron Communication in Sensory Ganglia; A Possible Role for Satellite Glial Cells During Chronic Pain

A large bulk of studies have implicated Connexin- and pannexin-based channels in several pathologies, mostly by an uncontrolled channel opening, which triggers increased release of metabolites such as ATP and ionic imbalance, with the consequent cell malfunction and eventually, cell lysis (Retamal et al., 2015). The mechanisms that control this enhanced open probability remain unclear. However, present evidence suggest that Connexin- and pannexin-channels open probability increases in response to changes in redox potential (Retamal, 2014), inflammatory cytokines (Orellana et al., 2013) and point mutations (Dobrowolski et al., 2007, 2008). Moreover, at the CNS aberrant hemichannel activity has been associated to astrocyte activation and posterior neuronal dysregulation (Orellana et al., 2012; Yi et al., 2016), likely due to an excessive release of gliotransmitters (Orellana et al., 2011; Torres et al., 2012). Accordingly, aberrant Cx43 expression and/or hemichannel malfunction in spinal cord astrocytes have been associated with pain related pathologies. For example, Cx43 increased expression has been associated with the maintenance of the late-phase of neuropathic pain in mice (Chen et al., 2012, 2014). Accordingly, the use of Gap26 -a Cx43 hemichannel blocker- attenuated the pain hypersensitivity in a mice cancer pain model (Li et al., 2017) and CORM-2—a CO donor and inhibitor of Cx43 hemichannels (León-Paravic et al., 2014) also decreased the levels of neuropathic pain in mice (Wang and Sun, 2017). Currently, the exact mechanism by which Cx43 hemichannels and/or GJCs present in spinal astrocytes are involved in pain-like behavior remains unknown. However, in a mice model of spinal cord injury, the activation of Sigma-1 receptor in astroglial endoplasmic reticulum (ER), induced astrocyte activation and Cx43 expression, which in turn caused mechanical allodynia (Choi et al., 2016). Thus, suggests that changes in the ER affect Cx43 expression and pain development/maintenance. Additionally, nerve injury induces TNF-α expression which in turn increases Cx43 hemichannel activity and chemokine release (Chen et al., 2014), suggesting that proinflammatory cytokines are involved in Cx43 enhancement in spinal cord astrocytes. Interestingly, Panx1 also participates in the CNS-associated pain responses, as the intrathecal administration of a Panx1 blocker (^10^Panx or probenecid) decrease the C-fiber activity and decrease mechanical hyperalgesia in a model of neuropathic pain in rats (Bravo et al., 2014).

Within the PNS, the notion that SGCs play an important role in the development and maintenance of chronic pain is well accepted (Adler et al., 2009; McMahon and Malcangio, 2009; Ohara et al., 2009; Ji et al., 2013; Hanani, 2015). We will analyze current evidence relating connexin and pannexin expression in SGCs and their association to pain.

## Trigeminal Ganglia

**Figure 1:**
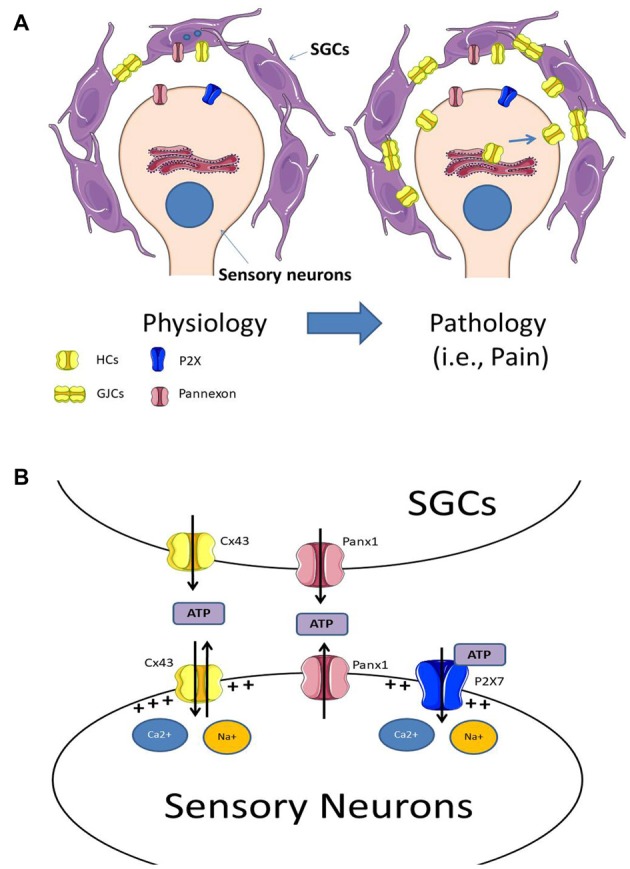
**(A)** Under physiological conditions sensory neurons are surrounded by satellite glial cells (SGCs), which are coupled by gap junction channels (GJCs). Experimental data indicates that SGCs express Panx1 and Cx43 hemichannels, and under physiological conditions, the neuro-glial communication through these channels may be low. Under pathological conditions (i.e., chronic pain) there is an increase in Cx43 expression in both SGCs and sensory neurons, augmenting both Cx43 hemichannel levels in their plasma membranes and the formation of GJCs between SGCs. **(B)** The increment of hemichannels and probably pannexons, lead to an increase of extracellular ATP. The elevated extracellular ATP concentration activates purinergic receptors at sensory neurons, which increase sensory neuron activity. Additionally, the increased activity of hemichannels and pannexons may also induce the depolarization of the plasma membrane due to an increase in the influx of Ca^2+^ and Na^+^.

Cherkas et al. (2004) reported that the axotomy of the trigeminal nerve increases both neuronal excitability and dye transfer between SGCs, as well as electrophysiological properties of both neurons and SGCs. Axotomy resulted in an increase in gap junctional communication between SGCs, which could also be associated to an increase of hemichannel activity. However, hemichannel activity in that model has not been tested yet. In agreement with the above-mentioned results, Cx43 expression in SGCs of the trigeminal ganglion was also increased in a rat model of chronic constriction injury (CCI; Ohara et al., 2008). Interestingly, the reduction of Cx43 expression using interference RNA reduced pain-like behavior in the CCI rats, but increased painlike behavior in non-CCI rats (Ohara et al., 2008), suggesting that Cx43 in trigeminal ganglion SGCs are involved in chronic pain (reviewed in, Ohara et al., 2009). Due to the lack of specific tools to discriminate between Cx43 GJCs and hemichannels, it is not possible to distinguish whether the increment of Cx43 expression seen in chronic pain models affects the expression of GJCs, hemichannels or both. An increase in Cx43 hemichannels at SGCs, may increase the release of gliotransmitters from these cells (Figure 1), facilitating the activation of neurons that may participate in the perception of chronic pain. The decrease in Cx43 by double strand RNA (dsRNA) in normal trigeminal neurons evoke the appearance of nociceptive responses similar to those seen following nerve injury (Jasmin et al., 2010), suggesting that, under normal conditions, the decrease of Cx43 may induce a lower K^+^ buffering capacity, which in turn increases neuronal excitability (Jasmin et al., 2010).

Unlike nerve damage, Cx43 levels in trigeminal SGCs do not change after acute (15 min–24 h) or chronic (3–7 days) inflammation using an injection of complete Freund’s adjuvant in the temporomandibular joint (Garrett and Durham, 2008). However, in such conditions, the levels of Cx26, Cx36 and Cx40 increased in both neurons and SGCs (Garrett and Durham, 2008). Similar results have been observed in a mouse model of chemotherapy-induced peripheral neuropathy (Poulsen et al., 2015), indicating that different pain models and/or different times of experimentation could induce different connexin expression patterns in the trigeminal ganglion.

SGCs could release some gliotransmitters that enhance the activity of sensory neurons. This idea is supported by an *in vitro* experiment, in which purinergic cross-talk signaling between SGCs and neurons was demonstrated. In this work they induced mechanical activation of SGCs which increased cytosolic Ca^2+^ in both neighboring SGCs and neurons. This intracellular Ca^2+^ rise was sensitive to P2X receptor antagonists and GJC blockers, suggesting that mechanical stimulation of SGCs induces hemichannel opening, allowing the ATP release, which is necessary to activate P2X receptors at SGCs and neurons (Suadicani et al., 2010). Additionally, the mechanic activation of neurons induced Ca^2+^ signaling in SGCs which was blocked by suramin but not by the GJC blocker carbenoxolone, suggesting that neuronal ATP release in a connexin-independent manner (Suadicani et al., 2010). The activity/signaling of these receptors can be affected by peripheral inflammation, as observed in primary cultures of trigeminal ganglion (Kushnir et al., 2011). In the paracrine communication between SGCs and sensory neurons, ATP appears as the most probable candidate, but the participation of other gliotransmitters such as glutamate cannot be ruled out, both of which may be released via hemichannels (Wagner et al., 2014).

## Nodose Ganglia

In the nodose ganglion, the intraperitoneal injection of LPS induced a 2-fold increase in the SGCs’ dye transfer, suggesting an increase in connexin expression and/or an increase in GJC activity. Interestingly, the authors found that Cx43 expression was decreased after 7 days of LPS, but there was an increase of Panx1 in both neurons and SCGs (Feldman-Goriachnik et al., 2015). In the nodose ganglion, Cx43 is expressed only in SGCs while Panx1 is expressed in SGCs as well as in neurons (Retamal et al., 2014a). The opening of hemichannels induced by incubation with extracellular fluid without Ca^2+^ and Mg^2+^ or with an agonist peptide called TATCx43CT (Ponsaerts et al., 2012) both increase the frequency of discharge of nodose neurons. The increased neuronal activity induced by the Ca^2+^-free culture media was partially inhibited by β-glycyrrhetinic acid (βGA) and a connexin 43 mimetic peptide (Gap27; Retamal et al., 2014a). Gap27 is a specific inhibitor of Cx43 hemichannels, suggesting that some gliotransmitters may have been released from SGCs via Cx43 hemichannels under these conditions.

## Petrosal Ganglia

To date, there is no available information about the presence and role of connexin and pannexin based channels in SGCs or neurons from the petrosal ganglion. However, a potential role has recently been proposed (Retamal et al., 2014b).

## Dorsal Root Ganglia (DRG)

Hanani et al. (2002) reported the first evidence showing SCGs in DRG responding to nerve injury. In this study, a section of a peripheral nerve (sciatic and saphenous) increased the number of GJCs between SGC surrounding a single neuron and between SGCs surrounding different neurons in response to nerve injury. Later, it was shown that nerve injury also increases the dye transfer between SGCs (Pannese et al., 2003). The techniques used in these studies are incapable of distinguishing which type of connexins was responsible for this increase in GJC size and gap junctional communication. However, in another study using a model in which the spinal cord was dissected or compressed at the T3, the levels of Cx43 in SGCs located in DRG at C6 to C8 were increased and the administration of mimetic peptides Gap19 or Gap27, specific blockers for Cx43 hemichannels reduced significantly tactile allodynia for 30 min after administration (Lee-Kubli et al., 2016), duration probably set by the short half-life of the peptides. This suggests that Cx43 hemichannels are potential candidates for pharmacological treatment of neuropathic pain. In another mouse pain model, partial colonic obstruction induced an increase of DRG neuronal activity due to a decrease of its resting membrane potential. Additionally, an increase of dye coupling between SGCs that are surrounding neurons was observed (Huang and Hanani, 2005). Furthermore, it was determined that neurons were not dye coupled neither to other neurons nor to SGCs (Huang and Hanani, 2005). These results suggest that in this pain model, there is not functional gap junctional communication between SGC and neurons. Later, the effect of colonic inflammation induced by local application of dinitrosulfonate benzoate upon gap junctional communication in DRG cells was studied (Huang et al., 2010). After the experimental procedure, increased neuronal activity and dye transfer between SGCs was observed. The higher increment in neuronal activity triggered by the procedure was suppressed by three different GJC blockers; carbenoxolone (50 μM), meclofenamic acid (100 μM) and palmitoleic acid (30 μM), suggesting that gap junctional communication is associated to the hyperactivity of sensory neurons (Huang et al., 2010). However, none of these blockers are specific for GJCs. Therefore, the possibility that hemichannels may participate in the release of gliotransmitters which enhance neuronal activity cannot be ruled out. Similar results have been observed in mouse models of experimental neuropathic autoimmune encephalomyelitis (Warwick et al., 2014), diabetes mellitus (Hanani et al., 2014), sepsis (Blum et al., 2017) and chemotherapy-induced peripheral neuropathy (Warwick and Hanani, 2013). In all these studies an increase in gap junctional communication between SGCs was observed, but the molecular mechanism behind this phenomenon remains unknown. However, a study performed in the sciatic nerve revealed that nerve transection induced an increase of dye coupling between SGCs, mostly by an increase in the number of GJCs (Ledda et al., 2009). On the other hand, the gap junctional communication between SGCs is affected by changes in K^+^, Ca^2+^ and pH (Huang et al., 2005), suggesting that GJCs between SGCs are modulated not only after neuronal injury, but they can also be modulated under physiological conditions.

In the case of pannexins the data available on their role is very limited. Only a handful of studies have investigated the role of pannexons in physiological conditions. One of these revealed that sensory neurons from DRG express Panx1 (Bele and Fabbretti, 2016). One of the most interesting findings in this work was that the activation of P2X3 receptor induced the activation of pannexons formed by Panx1, through a calcium/calmodulin-dependent serine protein kinase 3 (CASK)-dependent pathway. Once neuronal pannexons opened, massive ATP release and depolarization were detected (Bele and Fabbretti, 2016). Consistent with this finding, spinal nerve ligation increased Panx1 mRNA in DRG neurons, and the use of a siRNA against Panx1 decreased the hypersensitivity induced by nerve injury (Zhang et al., 2015). Moreover, in a mouse model of chronic orofacial pain, the selective deletion of Panx1 in GFAP-positive SGCs in the trigeminal ganglion eliminated the hypersensitivity and when Panx1 was deleted from sensory neurons, a reduction in baseline sensitivity was observed (Hanstein et al., 2016). The above studies suggest that Panx1 may participate in the modulation of neuronal sensory activity and therefore emerge as a possible novel target for the therapeutic treatment of chronic pain.

## Conclusion

As a general model of what is known so far, under control conditions, sensory neurons express mainly Panx1 and P2X receptors (i.e., P2X7) while SGCs express Cx43 and Panx1. After an insult, SGCs over-express Cx43, which in turn form additional GJCs and increase the number of functional hemichannels at their plasma membrane. Additionally, the insult also triggers the expression in sensory neurons of Cx43 hemichannels, but not GJCs, as they do not appear to form electrical synapses between them (for a scheme see Figure 1A). The increased expression of hemichannels and pannexons in sensory neurons and SGCs enhance extracellular ATP concentration, activating ATP-receptors such as P2X7. The activation of hemichannels, pannexons and P2X7 receptors finally induce the depolarization of the sensory neurons, which in turn, enhance their action potential firing rate (for a scheme, see Figure 1B).

Sensory neurons and SGCs express different connexin types, among them, Cx43 has been the most studied, being responsible for the formation of GJCs between SGCs. However, current evidence for functional hemichannels and pannexons is limited. It is however becoming clear that when hemichannels and/or pannexons open, there is an evident increase in extracellular ATP levels. The extracellular ATP generates Ca^2+^ oscillations in both SGCs and neurons, through P2X and P2Y receptors. Connexin-based hemichannels have been involved in a plethora of diseases (Retamal et al., 2015) and the sensory ganglia do not appear to be an exception. Thus, connexin-based channel blockers consistently decrease neuronal hyperactivity and pain-related behaviors in murine and rat models of pain/inflammation. Perhaps one of the most serious concerns about these results is that in most of the studies non-specific tools affecting or studying connexin-based channels were used. Therefore, it is not possible to dissect the role of hemichannels and GJCs. So far, it is clear that new tools are needed, such as specific and long half-life small molecules, antibodies or peptides that can dissect specifically the role of hemichannels and GJC (Riquelme et al., 2013). Until now, the use of connexin mimetic peptides has been the main tool for studying the acute role of hemichannels in the nervous system.

## Author Contributions

MARe, MARi, JS and JA wrote and edited this article.

## Conflict of Interest Statement

The authors declare that the research was conducted in the absence of any commercial or financial relationships that could be construed as a potential conflict of interest. The reviewer SV and handling Editor declared their shared affiliation.
